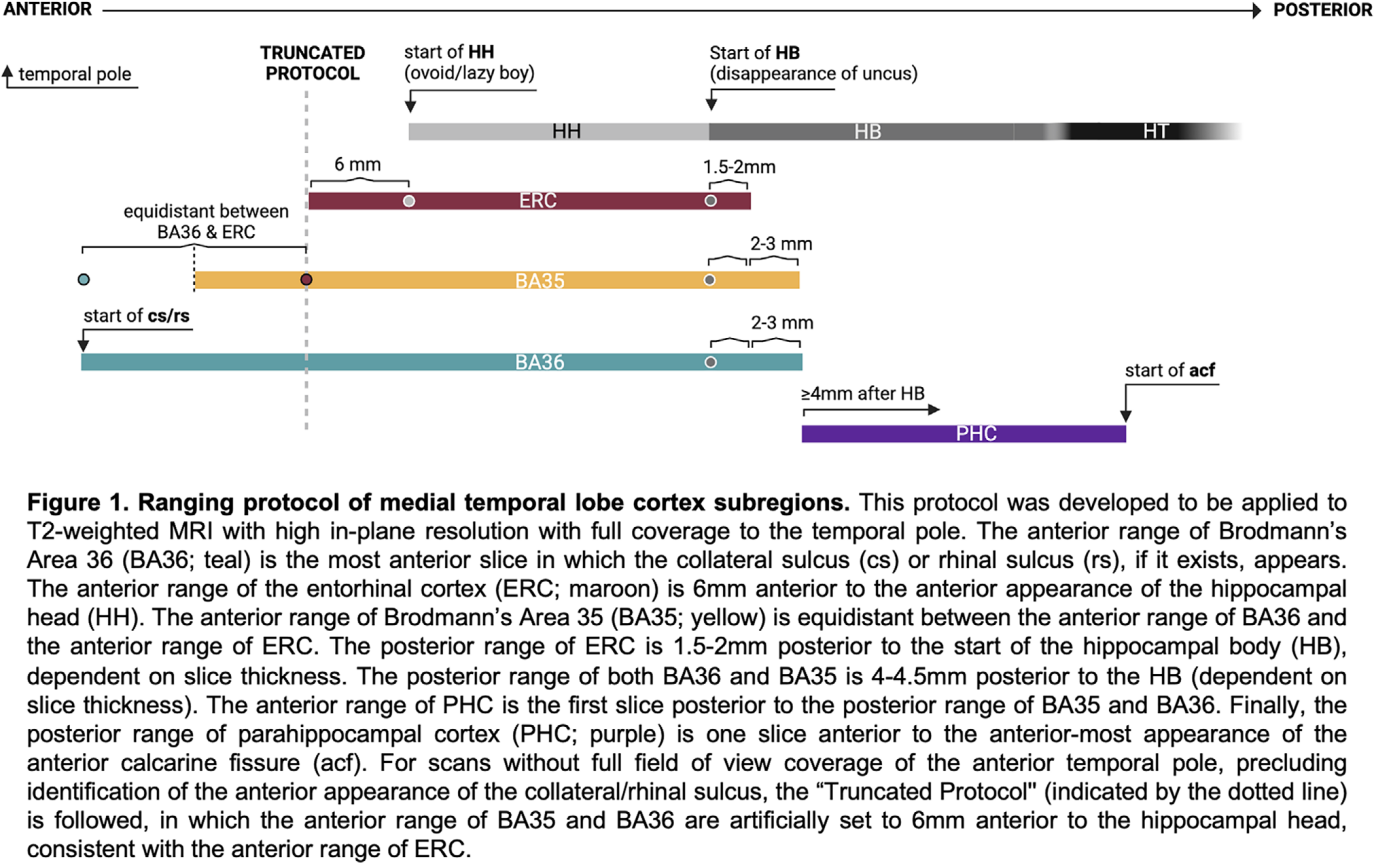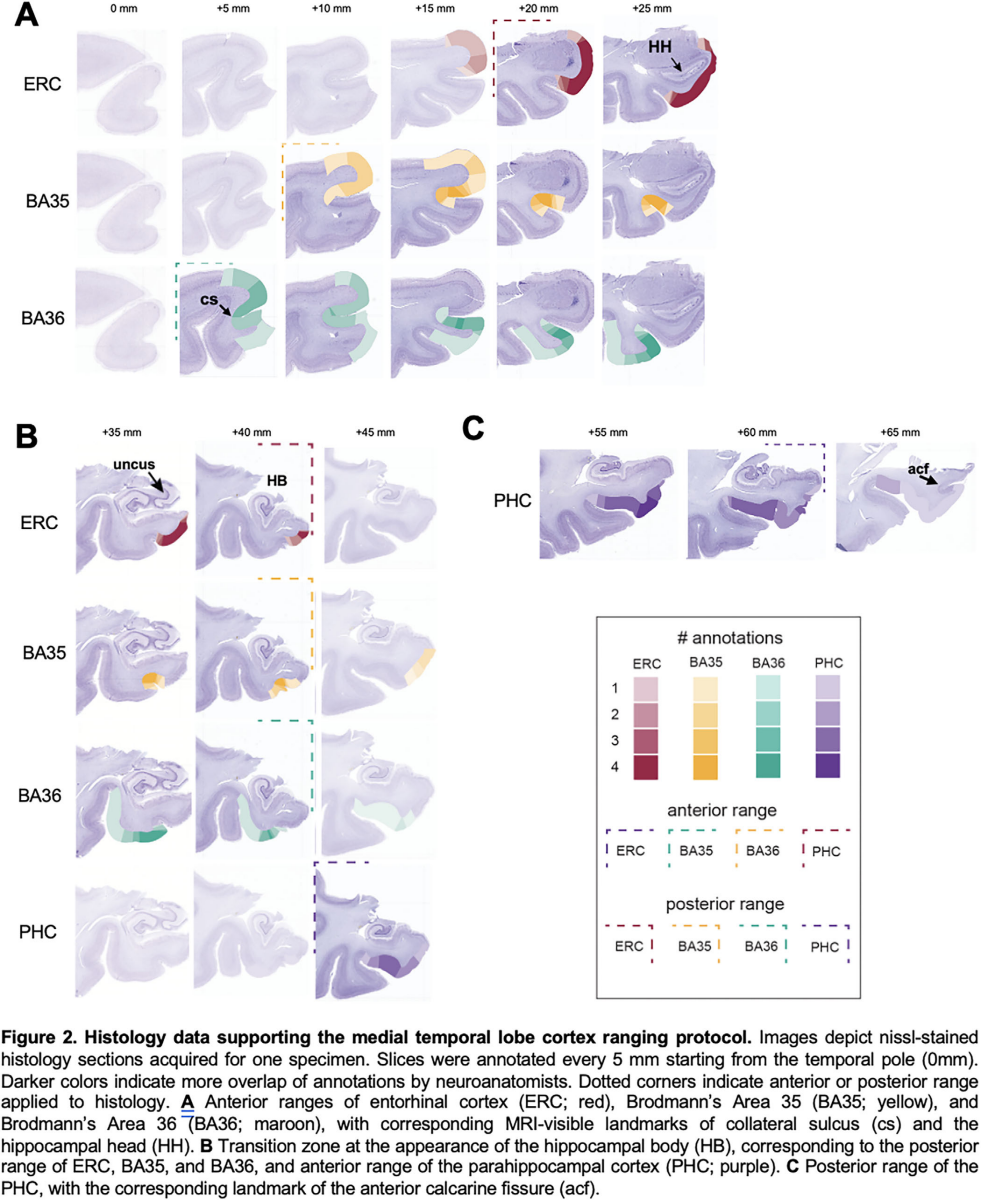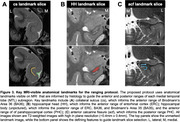# A harmonized, histology‐based protocol for selection of medial temporal lobe cortical subregion ranges on magnetic resonance imaging

**DOI:** 10.1002/alz.094891

**Published:** 2025-01-09

**Authors:** Jenna N. Adams, Hannah Baumeister, Thanh Doan, Anne Maass, Negar Mazloum‐Farzaghi, Tammy T Tran, Anika Wuestefeld, Jean Augustinack, Song‐Lin Ding, Ricardo Insausti, Olga Kedo, Arnold Bakker, David Berron, Kelsey L. Canada, Valerie A Carr, Marshall Axel Dalton, Ana M. Daugherty, Robin de Flores, Renaud La Joie, Susanne G Mueller, Rosanna K Olsen, Craig EL Stark, Lei Wang, Laura E.M. Wisse, Paul A. Yushkevich

**Affiliations:** ^1^ University of California, Irvine, Irvine, CA USA; ^2^ German Center for Neurodegenerative Diseases (DZNE), Magdeburg Germany; ^3^ Norwegian University of Science and Technology, Trondheim Norway; ^4^ University of Toronto, Toronto, ON Canada; ^5^ Rotman Research Institute, North York, ON Canada; ^6^ Stanford University, Stanford, CA USA; ^7^ Clinical Memory Research Unit, Lund University, Lund Sweden; ^8^ Martinos Center for Biomedical Imaging, Massachusetts General Hospital, Harvard Medical School, Charlestown, MA USA; ^9^ Allen Institute for Brain Science, Seattle, WA USA; ^10^ University of Castilla‐La Mancha, Albacete Spain; ^11^ Forschungszentrum Jülich, Julich Germany; ^12^ Department of Psychiatry and Behavioral Science, Johns Hopkins University School of Medicine, Baltimore, MD USA; ^13^ Institute of Gerontology, Wayne State University, Detroit, MI USA; ^14^ San Jose State University, San Jose, CA USA; ^15^ The University of Sydney, Sydney, NSW Australia; ^16^ Wayne State University, Detroit, MI USA; ^17^ Normandie Univ, UNICAEN, INSERM, U1237, PhIND “Physiopathology and Imaging of Neurological Disorders”, NeuroPresage Team, GIP Cyceron, Caen France; ^18^ Memory and Aging Center, Weill Institute for Neurosciences, University of California, San Francisco, San Francisco, CA USA; ^19^ Department of Radiology, University of California, San Francisco, San Francisco, CA USA; ^20^ Center for Imaging of Neurodegenerative Diseases, San Francisco, CA USA; ^21^ Rotman Research Institute, Toronto, ON Canada; ^22^ Department of Neurobiology and Behavior, University of California, Irvine, Irvine, CA USA; ^23^ Ohio State University Wexner Medical Center, Columbus, OH USA; ^24^ Department of Clinical Sciences Lund, Lund University, Lund, Lund Sweden; ^25^ University of Pennsylvania, Philadelphia, PA USA

## Abstract

**Background:**

The medial temporal lobe (MTL) has distinct cortical subregions that are differentially vulnerable to pathology and neurodegeneration in diseases such as Alzheimer’s disease. However, previous protocols for segmentation of MTL cortical subregions on magnetic resonance imaging (MRI) vary substantially across research groups, and have been informed by different cytoarchitectonic definitions, precluding consistent interpretations. The Hippocampal Subfields Group aims to create a harmonized, histology‐based protocol for segmentation of MTL cortical subregions that can reliably be applied to T2‐weighted MRI with high in‐plane resolution.

**Method:**

Nissl‐stained sections from the temporal lobes of three human specimens (66‐90 years old; 2 female) were annotated by four expert neuroanatomists for the following MTL subregions: entorhinal cortex (ERC), Brodmann’s Area 35 (BA35; largely corresponding to “transentorhinal” cortex), Brodmann’s Area 36 (BA36), and parahippocampal cortex (PHC). On each histology section, the number of annotations and the spatial overlap of annotations were analyzed to determine the consensus of the anterior to posterior range of each structure. Gross anatomical landmarks, detectable on MRI and reliably corresponding with each range, were then selected to create an MRI ranging protocol. Feasibility of this MRI protocol was tested by two independent raters across four MRI scans (two healthy adults, two older adults), and agreement in range selection was assessed using Cohen’s kappa statistic.

**Result:**

The proposed MTL ranging protocol is shown in **Fig. 1**, and corresponding histology data substantiating the protocol is shown in **Fig. 2**. MRI‐visible gross anatomical landmarks that reliably corresponded with the anterior or posterior range of each subregion on histology included the anterior‐most appearance of the collateral sulcus (**Fig. 3A**), hippocampal head (**Fig. 3B**), hippocampal body, and anterior calcarine fissure (**Fig. 3C**). This protocol demonstrated high feasibility when applied to MRI, with average kappa values of 0.75 ± 0.07, representing a “substantial” level of agreement of range selection.

**Conclusion:**

Future directions include obtaining consensus on this protocol from the larger research community through a Delphi procedure, and expansion of the protocol to include slice‐by‐slice segmentation guidelines for full delineation. This harmonized, histology‐based protocol will facilitate critical research on MTL subregion vulnerability and their contributions to memory deficits in Alzheimer’s disease.